# Huntington Disease as a Neurodevelopmental Disorder and Early Signs of the Disease in Stem Cells

**DOI:** 10.1007/s12035-017-0477-7

**Published:** 2017-05-11

**Authors:** Kalina Wiatr, Wojciech J. Szlachcic, Marta Trzeciak, Marek Figlerowicz, Maciej Figiel

**Affiliations:** Institute of Bioorganic Chemistry, Polish Academy of Sciences, Noskowskiego 12/14, 61-704 Poznan, Poland

**Keywords:** Stem cells, Polyglutamine diseases, ESC, iPS, iPSC, NSC, Neurodegenerative disease, Neurodevelopmental disease, polyQ disease, Huntington disease

## Abstract

Huntington disease (HD) is a dominantly inherited disorder caused by a CAG expansion mutation in the huntingtin (HTT) gene, which results in the HTT protein that contains an expanded polyglutamine tract. The adult form of HD exhibits a late onset of the fully symptomatic phase. However, there is also a long presymptomatic phase, which has been increasingly investigated and recognized as important for the disease development. Moreover, the juvenile form of HD, evoked by a higher number of CAG repeats, resembles a neurodevelopmental disorder and has recently been the focus of additional interest. Multiple lines of data, such as the developmental necessity of HTT, its role in the cell cycle and neurogenesis, and findings from pluripotent stem cells, suggest the existence of a neurodevelopmental component in HD pathogenesis. Therefore, we discuss the early molecular pathogenesis of HD in pluripotent and neural stem cells, with respect to the neurodevelopmental aspects of HD.

**Electronic supplementary material:**

The online version of this article (doi:10.1007/s12035-017-0477-7) contains supplementary material, which is available to authorized users.

## Introduction

The onset of serious motor and cognitive symptoms is late in Huntington disease (HD). However, extensive longitudinal research in the PREDICT-HD [1] and other clinical programs [2] indicates that premanifest HD patients develop subtle HD-mediated changes decades before classical diagnosis. These changes include cognitive, functional, and psychiatric symptoms; altered brain morphology and connectivity; and even subtle motor deficits [3–8]. Moreover, neuronal degeneration and the deregulation of neurodevelopmental genes occur long before the onset of classical HD symptoms and phenotypic changes in both mouse models and patients [9–14]. These findings raise the question of whether premanifest symptoms in HD are a consequence of neurodevelopmental abnormalities. Neurodevelopmental deficits typically occur in childhood. For instance, children at risk of HD exhibit smaller head size, indicating a deficit in brain growth [15]. In addition, lower body weight index (BMI) is also present in prodromal HD children and body weight deficit was identified for juvenile HD suggesting a developmental deficit probably due to mitochondrial dysfunction [16]. The HD juvenile form, also known as “Westphal variant,” is characterized by a high number of CAG repeats, onset under 20 years of age, and disease manifestation different than that of adult-onset disease [17, 18].

**Fig. 1:**
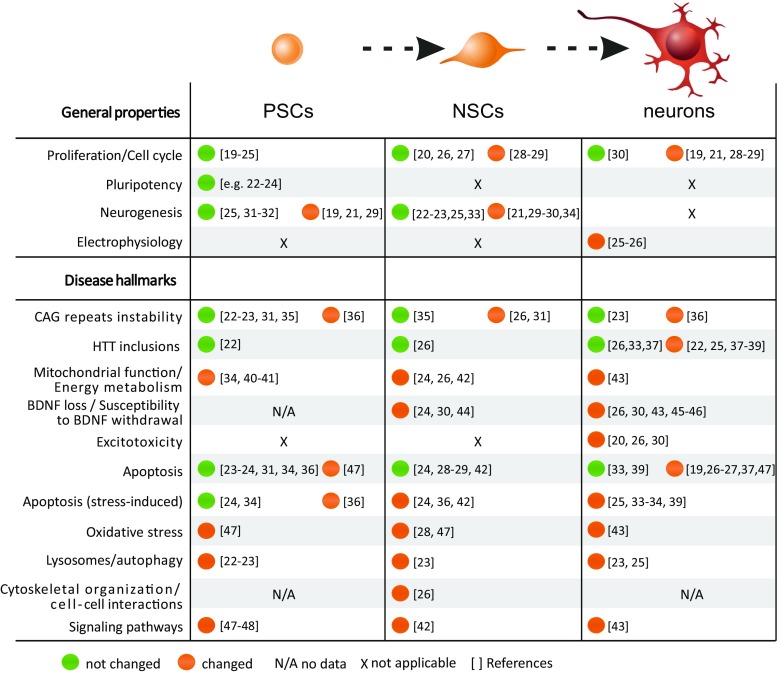
HD pathogenic changes occur along the differentiation axis: pluripotent stem cells → NSC → mature neurons. The figure summarizes HD pathogenesis in the indicated cell types. Many HD-associated changes are present in the early stages of development, beginning with pluripotent stem cells and continuing throughout differentiation to NSC and mature neurons. The data form high-throughput transcriptomics and proteomics were not included in the table. Changes detected (*orange*), absence of change (*green*), discrepant results (*gray*), data non-available (*N/A*), and non-applicable (*X*) (Color figure online)

Multiple roles have been established for normal and mutant huntingtin (HTT) in pre- and postnatal development via in vivo and in vitro developmental research on animal models. Evidence from recent stem cell studies supports the idea that mutant HTT-dependent changes may be detected early, even at the naïve pluripotent cell stage (see discussion and references in the following sections). Therefore, it is important to elucidate the pathogenesis of HD along the differentiation axis (pluripotent stem cells (PSC) → neural stem cells (NSC) → mature neurons, Fig. 1) to identify the early processes relevant to developmental defects and disease onset. Understanding the pathogenesis in these cell types is particularly important for the development of effective cell therapies and determination of the therapeutic impact.

The aim of this work is to review the current knowledge regarding the most important facts about the role of normal and mutant HTT in organism development. Subsequently, we will discuss early molecular pathogenesis of HD identified in PSC and NSC, which may underlie the developmental deficits in HD. Additionally, we have performed a meta-analysis to determine whether previously identified deregulated genes/proteins in HD PSC, NSC, and neurons are related to developmental Gene Ontology (GO) terms.

### HTT Is Necessary for Development

**Fig. 2:**
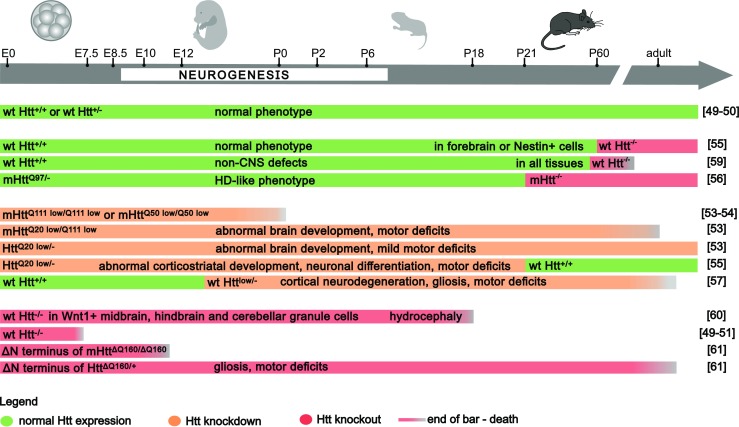
Phenotypic effects of the HTT gene manipulation during mouse development. The diagram illustrates the knockout and knockdown of normal and mutant HTT, at several points in time of mouse embryonic and postnatal development, indicating CNS and non-CNS consequences. The mouse age and the duration of HTT knockout (*red bars*) or knockdown (*orange bars*) in mouse life are indicated by the *relevant length* of *bars*. *Green* indicates non-manipulated, wild-type HTT expression. Premature death or embryonic death is indicated by a *shorter bar*, ending with a *gray-colored gradient*. The phenotypic effects of the mutant and WT HTT gene manipulation are described directly on the *bars*. The mutant and WT HTT gene manipulation is indicated as genotype for each experiment at the beginning of each segment of the *bar* (Color figure online)

HTT is crucial for the organism development (Fig. 2), and the absence of HTT is lethal in mice around developmental day E7.5–8.5 [49–51]. This lethality may be caused by early embryonic patterning deficits, including shortened primitive streak and lack of headfolds, which denote a failure in the development of the head [52]. The expression of wild-type HTT at approximately 10–15% of normal levels may rescue this embryonic lethality; however, the HTT insufficiency causes abnormal brain development and mild movement abnormalities [53]. On the other hand, similar low levels of mutant Q50 or Q100 HTT lead to perinatal death [53, 54]. The discrepancy in the effects of low levels of wild-type vs mutant HTT may result from both the loss of function of mutant HTT during development and/or the protective role of normal HTT, interfering with the gain of function of mutant HTT. The decreased level of wild-type HTT (10–15% of normal levels) in mouse embryos, followed by reconstitution of HTT expression to normal level on postnatal day 21, resulted in progressive striatal and cortical neuronal degeneration and motor incoordination later in life [55]. In addition, when mice are exposed to normal levels of mutant HTT 97Q until postnatal day 21, they develop a HD-like phenotype including neuropathology and motor deficits. The phenotype is not as severe as in the mice with lifelong expression of HTT 97Q [56]. Summarizing, low level of HTT or expression of mutant HTT, limited to the time of embryonic development and short postnatal time when striatal neurogenesis occurs, is sufficient for generation of the neurological phenotype in mice. In addition, the conditional reduction of HTT in the mouse forebrain, which is initiated at later embryonic stages and reached 84% reduction of expression by postnatal day 60, leads to progressive neurodegeneration and premature death [57]. In addition, the phenomenon called “huntingtin holiday” also suggests that the disease symptoms in HD may be reversed but the reversal is not complete [58]. HTT knockout (KO) in Wnt1-expressing cells of the midbrain, hindbrain, and cerebellar granule cells results in hydrocephaly and death at postnatal P6–18 [60]. In contrast, mice remained unaffected when HTT was knocked down in the forebrain or Nestin-positive cells at 2, 4, or 8 months of age [55]. HTT knockdown to the level hardly identified by immunoblotting in all mouse tissues at 2 months of age leads to death as a result of non-CNS defects, such as acute pancreatitis [59]. However, no neuronal deficits were identified in these mice. In addition, the deletion of polyglutamine (polyQ) or proline-rich regions within the N-terminus of HTT did not affect normal mouse development, whereas the N-terminus alone is insufficient to rescue lethality in embryonic and young mice [61–63].

### Developmental Functions of HTT

The neural rosettes are radial arrangements of cells in the culture, indicating that embryonic stem cells (ESC) differentiate and form NSC. Therefore, neural rosettes in culture are a developmental marker resembling the radial arrangements of NSC forming neural tube during development [64]. Mouse ESC-derived NSC with low expression of HTT are able to form rosettes; however, NSC which are deprived of HTT (HTT-null cells) are unable to form neural rosettes in vitro [65]. The phenotype, which is referred to as rosetteless, is reflected in the impaired acquisition of proper polarity during neurulation in HTT-null zebrafish embryogenesis [65]. It is a consequence of defective cell adhesion function of HTT, which depends on the N-terminal portion of the HTT protein, and is mediated by ADAM10/N-cadherin [65]. The cell adhesion function of N-terminus of HTT is a recent evolutionary step which probably enabled more complex development of the CNS [65].

HTT is essential for the formation and orientation of a proper mitotic spindle [66]. Its depletion during embryonic cortical neurogenesis by in utero electroporation, using HTT siRNA, causes incorrect spindle orientation, which results in a decreased pool of proliferating progenitors and increased differentiation due to an imbalance in symmetric vs asymmetric divisions [66, 67]. Similarly, the expression of mutant HTT in the absence of normal HTT in cells derived from Hdh^Q111/Q111^ mice causes mitotic spindle misorientation along with defects in the proliferation of neuroprogenitors [68].

Conditional reduction of HTT (less than 10% of the normal level), occurring selectively in cortical excitatory Emx1-expressing neurons, produces low HTT expression already at E 9.5, prior to early postnatal synaptic development. Notably, the depletion also includes cortical layer 5, which projects to the striatum. Such experimental setup demonstrated altered cortical and corticostriatal connectivity and the increase in excitatory synapse formation in the striatum, which suggests a non-cell-autonomous effect on maturation of striatal medium spiny neurons (MSNs) [69]. Similar changes have been identified in the corticostriatal development of HD knock-in zQ175 mice, which suggests HTT loss of function in the development of corticostriatal synaptic connectivity [69]. Aberrant cortical inputs may affect the proper maturation of striatal MSNs, since generation of striatal neural progenitors (NPC) is also compromised in HdhQ111 knock-in mice [70] and patients [71]. Abnormal specification and maturation of MSNs impair the acquisition of the proper mature striatal cytoarchitecture. Improperly matured MSNs may be vulnerable to stress-mediated cell death in the symptomatic stages of the disease. The overall evidence indicates a neurodevelopmental stage in HD and its significant role in the disease development.

## Considerations for HD Modeling in PSC and NSC

The earliest molecular phenotypes of HD pathology were identified in pluripotent stem cellular models. PSC recapitulate the cellular stages occurring during early stages of organism development. ESC are isolated from inner cell mass of the blastocysts, whereas the induced pluripotent stem cells (iPSC) are produced by cellular reprogramming of somatic cells, with the use of genetic mechanism described by Takahashi and Yamanaka [72]. Table 1 summarizes and provides an overview of the existing animal and HD patient stem cell models. This overview indicates that the existing HD stem cells possess highly variable characteristics, such as technology (derivation method, cell source, and epigenetic status), disease (number of CAG repeats), and experimental design (e.g., the number of lines directly compared in order to determine the phenotypes, and the lines compared had uniform or differing genetic backgrounds, i.e., isogenic or non-isogenic/mutation silenced/corrected). For example, the majority of HD patient iPSC were produced using stable genomic integration of reprogramming factors, which remain in iPSC genome after the reprogramming process and may affect the observed HD changes. In contrast, the newest techniques use the reprogramming systems which involve significant control over the expression of exogenous reprogramming factors and prevent integration of these factors with the genome of somatic cells during the reprogramming process. Such systems, e.g., based on Sendai virus, messenger RNA (mRNA), or episomal vectors, are denoted as integration free [73]. In addition, for identification of the phenotypes, iPSC from HD patients were compared to iPSC from other, genetically unrelated, non-affected patients. This type of comparison is characterized by a limited resolution due to genetic modifiers, which may affect phenotypes. For example, the CAG tract length in HTT is not entirely correlative with the disease onset and duration, pointing to the existence of additional genetic modifiers [91, 92]. HD phenotypes can also be modified by the epigenome states of iPSC, which may convey transcriptional characteristics of source somatic cells. Importantly, the epigenome states in iPSC are related to different types of pluripotency, namely primed and naïve pluripotency [93]. The currently available human iPSCs commonly occur as primed pluripotent cells, and such cell state first appears in later blastocyst stages [94]. Naïve pluripotent state is characteristic of cells of the inner cell mass of the early blastocyst, and such stage is typical of mouse ESC and iPSC. Naïve pluripotent cells self-renew rapidly and almost indefinitely in vitro, and their self-renewal is dependent on the leukemia inhibitory factor (LIF) [93]. An epigenetic state of both naïve and primed iPSC may be recognized by the methylation status of their genome. Naïve PSC have transcriptionally active hypomethylated genome, while the primed PSC already exhibit some methylation patterns characteristic of more advanced stage of the cell linage [93]. The available protocols for generation of human iPSC can be used to reprogram somatic cells until primed stage but are not suitable for direct reprogramming to developmentally earlier, naïve cell stage characterized by fewer epigenetic marks. Therefore, the genome of primed human iPSC exhibits remaining epigenetic marks of source cells and additionally acquires new epigenetic marks by reprogramming or continuous culture [95, 96]. Such epigenetic marks can affect the differentiation potential and development of HD phenotypes [80].

**Table 1:** Pluripotent models of HD

Species and cell type	Name (mutQ number)	Model/cells of origin	Derivation method (factors)	Isogenic	References
Mouse ESC	Hdh CAG150 (150)	Hdh CAG150 knock-in	Embryo	Syngeneic	[19]
	*Hdh* ^*Q20/7*^ (20) *Hdh* ^*Q50/7*^ (50) *Hdh* ^*Q91/7*^ (91) *Hdh* ^*Q111*/7^ (111)	129Sv mES	Knock-in	Yes	[40] [21, 27, 29, 32]
	*Htt* ^*F140Q/7Q*^	mES	Knock-in	Yes	[28]
	HD ESC (127)	R6/1-HTTex1	Embryo	Syngeneic	[36]
	*Hdh* ^*Q20/7*^ (20) *Hdh* ^*Q140*/7^ (140)	CAG140 knock-in	N/d	Syngeneic	[27, 41]
Monkey ESC	TrES1 (72) [tetraploid]	rHD-HTTex1-GFP fibroblasts/WT oocyte	Hybrid embryo	No	[73]
	rHD-ES—7x (28–48, one line 131)	rHD-HTTex1-GFP	Embryo	No	[39, 74]
Human ESC	hESC-184 hESC-196	IVF/PGD^b^	Embryo	No	[75, 76]
	VUB05_HD (44)	IVF/PGD^b^	Embryo	No	[35, 76, 77, 78 ]
	SI-186 (37) SI-187(51)	IVF/PGD^b^	Embryo	No	[31]
	STR-155-HD (44) a.k.a. Huez2.3	IVF/PGD^b^	Embryo	No	[79, 76, 78]
	SIVF017-HD (40) SIVF018-HD (46) SIVF020-HD (48) SIVF046-HD (45) a.k.a. GENEA017,18,20,46	IVF/PGD^b^	Embryo	No	[80, 34, 76]
	Q23 (23) Q73 (73) Q145 (145)	H9 hESC	piggyBAC transposon w/ HTTex1 cDNA	Yes	[37]
	GENEA089 (41) GENEA090 (46) GENEA091 (42)	IVF/PGD^b^	Embryo	No	[34]
	KCL027 (43) KCL028 (43) KCL036 (38)	IVF/PGD^b^	Embryo	No	[81]
Mouse iPSC	HD-iPS (two mice) (144)	R6/2-HTTex1 transgenic fibroblasts	Retroviral (OSKM)	Syngeneic	[22]
	YAC-HD-iPS (128)	YAC128 transgenic fibroblasts	piggyBac (OSKML), excised from genome	Syngeneic	[48]
Monkey iPSC	RiPS-3 (72) +3 other	rHD-HTTex1-GFP fibroblasts	Retroviral (OSK)	No	[82]
	HD-14 (27/65)	rHD-HTTex1-GFP dental pulp stromal cells	Retroviral (OSK)	No	[39]
Human iPSC	HD-iPS-4 (GM23225) and HD2 (72)	Fibroblasts: GM04281	Retroviral (OSKM)	No	[83] [24, 38, 42, 44, 47, 84]
	Corrected-HD-iPS4 clones C127 (21) and C116 (20)	HD-iPS4 (72) line	Homologous recombination	Yes	[42]
	HD-iPS^HOM^ 4F/3F (42/44)^c^ HD-iPS^HOM^ 4F (39/43)^c^ HD-iPS^HET^ 3F (45)	Fibroblasts: HD509 HD832 HD1657	Lentiviral (OSKM/OSK)	No	[23]
	F-HD-iPSC (50) D-HD-iPSC (109)	Fibroblasts	Retroviral (OSKM)	No	[85]
	HD60i (60) HD109i.1 (109) HD180i (180)	Fibroblasts: GM03621 ND39258 GM09197	Lentiviral (OSKMNL) Retroviral (OSKM)	No	[26]
	HD1—5x (n/d) HD2—5x (86Q)	Fibroblasts: GM04693 GM05539	Lentiviral (OSKM)	No	[43]
	HD70 (70) HD180 (180)	Fibroblasts: GM21756 GM09197	Episomal (OSKML+sh-p53)	No	[86, 87]
	HD60n (60) HD109n (109) HD180n (180)	Fibroblasts: GM09197 ND39258 GM03621	Episomal (OSKML+sh-p53)	No	[30]
	ND4228, ND4229, ND4230 (71) ND4222, ND4223, ND4224 (109)	Fibroblasts: GM04281	Episomal (OSKML+sh-p53)	No	NINDS^a^ [48]
	ND41657 (57)	Fibroblasts: ND33392	Episomal (OSKML +sh-p53)	No	NINDS^a^ [88, 89]
	HD-iPSC-A1, −A7 (43) HD-iPSC-B4, −B16 (43)	Fibroblasts	Retroviral (OSKMN)	No	[33]
	Q47 (47) Q70 (70)	Fibroblasts	Lentiviral (OSKM)	No	[46]
	iPSHD11 (40) iPSHD22 (47) iPSHD34 (42)	Fibroblasts	Lentiviral (OSKM)	No	[25]

^a^These cell lines have been deposited at the National Institute of Neurological Disorders and Stroke (NINDS) repository, https://stemcells.nindsgenetics.org, and were obtained from there by authors of the cited articles

^b^IVF/PGD—embryos donated after preimplantation diagnostics of in vitro fertilization procedure

^c^These lines were derived from a homozygous patient

One potential solution to overcome variability in determining HD phenotypes is to increase the statistical power by increasing the number of patients and iPSC lines. However, the best solution is to employ genetic technologies, such as genome editing or constant short hairpin RNA (shRNA) expression, to generate corrected, patient-specific isogenic cells suitable for use as control cell lines. A complementary solution is to use the iPSC from healthy patients, to generate the isogenic iPSC with incorporated mutant genes. Therefore, the current knowledge of HD-mediated changes will have to be verified using more technologically advanced stem cell systems.

NSC are multipotent, self-renewing cells that differentiate into neural, glial, and oligodendrocyte lineages and represent the next stage of differentiation in the process of nervous system development. In the adult mammalian brain, NSC contribute to the brain plasticity and are located in the dentate gyrus of the hippocampus and the telencephalic subventricular zone [98]. During postembryonic and embryonic neurogenesis, NSC undergo symmetric and asymmetric divisions to generate NPC, which are unipotent or oligopotent; thus, they have a limited ability to self-renew and are committed to neural fate. Both NSC and NPC require growth factors and extracellular signals to regulate proliferation and differentiation. NSC may be maintained in vitro, in multiple, distinct cellular stages of nearly homogenous cells, which may reflect the ongoing transitional developmental progress of their in vivo counterparts [99, 100]. Notably, these distinct expandable states are composed of previously established, selected cell populations, and end-point analyses of these populations may not capture events relevant to step-wise in vivo development. Therefore, for modeling a disease, NSC and NPC should be individually derived from PSC for each experiment, as this method might be more accurate than using the high-passage NSC.

### Pluripotency and Self-Renewal

HTT is not required for the maintenance of a pluripotent state in mouse cells [101]. Additionally, HD mutation does not influence iPSC generation or other features of the pluripotent state, including the expression of pluripotency network genes and the general ability to differentiate into cells that originate from all germ layers [22, 23, 26, 48].

The self-renewal and cell cycle of NPC are altered in the developing and adult HD mice, as well as postmortem HD patient brains [66, 70]. However, no differences have been identified in the proliferation and cell cycle of human HD ESC/iPSC or derived NSC [21, 23, 24]. In contrast, lower proliferation rates have been identified in heterozygous (ESC-derived) and homozygous (brain-derived) NSC from HD 140CAG KI mice vs WT NSC [28]. At another laboratory, heterozygous NSC from the same mice did not exhibit differences in the proliferation or cell cycle, despite similar culture conditions [27]. Similarly, there were no differences between isogenic HD Q20, Q50, and Q111 NSC [27]. These studies used established, high-passage monolayer NSC cultures, which represent a homogenous population of NSC. In contrast, Nguyen et al. used a step-wise neuralization protocol, with NSC analyzed at each step, and they identified an increased proliferation of HD Q111 ESC-derived NSC [29]. Therefore, the discrepant effect of full-length mutant HTT on cell growth characteristics using mouse cell lines may be the result of several variations between cell lines, such as differential culture protocols, exact source of stem cells, and developmental timing of cell isolation.

### Differentiation Potential

HTT is required for NSC rosette formation [65]; however, its loss does not affect the derivation and identity of postrosette, radial glia-like, NS populations [27, 102]. Interestingly, pre-rosette [26, 30], rosette [24, 42] and NS states [27] may be derived and maintained in HD cells. Reports by Nguyen and colleagues, which are more closely focused on cellular identity transitions, have indicated that both the loss and mutation of HTT in mouse ESC impair the specification and maturation of progenitors within all germ layers [21, 29]. Mutant HTT has been shown to promote neuroectodermal fate (increased numbers of Sox1+ and Nestin+ cells) and advanced neuronal maturation, as well as increased acquisition of oligodendrocyte fate, at later stages of NSC maturation.

The iPSC derived from transgenic R6/2 mice did not present overt neural differentiation deficits [22]. In contrast, differentiation of ESC from mouse HD 150Q knock-in resulted in more neural precursors (Sox3+) and neurons (β-tubulin+), than differentiation of WT NSC [19]. Similar results were obtained from adult HD 150Q NSC isolated from SVZ [19]. Furthermore, an increase has been observed in population of Nestin+ NPC after 42 days of differentiation of human juvenile HD iPSC (60, 109, and 180Q) towards striatal-like fates [30]. Interestingly, this retained Nestin+ population, and not mature neuronal cells, appeared to account for the previously shown population of striatal-differentiated juvenile HD cells susceptible to excitotoxic death induced by BDNF withdrawal [26]. BDNF is deficient in HD [103] and it is an important regulator of adult neurogenesis [104]. Therefore, the results indicate that the deficit in BDNF affects immature neuronal progenitors, rather than mature cells, possibly reducing a pool of endogenous cells, which may be able to regenerate the affected brain regions. Two additional groups have reported similar findings, with decreased neural differentiation using a paradigm for efficient differentiation of late-passage, adherent mouse NSC into matured GABAergic neurons [105]. In one report, homozygous, mouse HD Q140 knock-in NSC resulted in fewer Nestin+ NPC, fewer βIII-tubulin+ neurons, and more Gfap+ glial cells than WT cells [28]. Similarly, fewer neuronal cells (Map2+ and Tau+) were identified in HTT KO cells and heterozygous Q20–140 knock-in cells [27]. However, increases in glial populations (Gfap2+ and S100β+) were only present in KO cells, which suggests that these putative loss-of-function mechanisms may be rescued by the mouse WT HTT allele in heterozygous HD cells. GABAergic neurons, which are mostly affected in HD, have not been directly assessed in the previously described studies; however, a decreased acquisition of GABAergic cell fate was evident in KO and Q111 mouse ESC [21]. In addition, a decreased neurite length was demonstrated in GABAergic neurons that originated from juvenile human HD iPSC (86Q) [43]. In contrast, the differentiated human ESC displayed an increased number of GABAergic neurons, increased number of neural nodes, but no change in neurite length [34]. Additional studies using human ESC and iPSC with lower adult HD CAG numbers indicated no deficit of maturation into GABAergic projection neurons [20, 23, 25, 33], which suggests greater developmental deficits in GABAergic maturation in the case of juvenile-onset HD than in case of adult-onset HD.

Evidence from investigation of differentiation of HD pluripotent models, together with the brain region-specific effects of HTT identified in vivo in mice and patients, indicates that the true effects of mutant HTT may sometimes be masked in artificial cell culture systems. Therefore, researchers should consider using conditions closer to an in vivo situation, e.g., cerebral organoids, to delineate the developmental deficits in HD more precisely, using adult and juvenile human HD cells.

### CAG Number and Genomic Stability

In HD the CAG length is dynamic and may undergo changes during gametogenesis and in somatic cells [106, 107]. The length of the parentally transmitted mutation correlates with the disease onset and severity. However, progression of the disease can be modified by somatic expansions. For instance, vast somatic expansions were identified in premanifest and late-phase, postmortem brains [108]. Large expansions in HD stem cells may change the interpretation of research data, since the length of the mutation might influence the severity of the cellular phenotype. The expansion rate in mice is cell-type dependent and occurs in postmitotic neurons as a consequence of a defective DNA repair of stress-induced DNA breaks [107, 109]. ESC and NSC are characterized by an increased DNA damage response and repair systems, which are essential for their developmental roles [110]. Therefore, the absence of changes [23, 27, 35] or mild [26, 31] changes in the CAG length in human and mouse pluripotent and neural HD stem cells is not surprising. Following differentiation, HD cells do not undergo overt repeat instability for up to 10 weeks. This was assayed in vitro in neuronal [23] and non-neuronal lineages (cardiomyocytes) [90], as well as in vivo in teratomas [35, 90]. The main exception to the previously described research includes data obtained from R6/1 mouse-derived ESC, in which repeat instability was identified in both pluripotent and neural differentiated states [36]. On average, there was a threefold instability increase when cells were challenged by peroxide-induced oxidative stress. Importantly, peroxide induced the upregulation of specific DNA repair system genes compared with WT cells. These findings suggest that increased susceptibility and reaction to stress-induced DNA are features of HD PSC, compared with non-affected lines.

The overall rate of genomic mutations is lower in PSC than in somatic cells; however, the reprogramming process and prolonged in vitro self-renewal conditions increase the chances of acquiring mutations [111]. Human HD iPSC have an increased rate of genomic instability during reprogramming, when p53 silencing is used in the process [96]. Reprogramming evokes increased replication stress [112] as a result of genome reorganization and extensive proliferation, which may explain the increased rate of CAG expansion identified in the reprogrammed HD iPSC [95]. However, in a long-term cell culture, the genomic integrity of iPSC remained unaffected by the mutant *HTT* gene [23, 96].

These experiments indicate that CAG instability in HTT gene is limited in PSC and NSC in vitro. Moreover, mutant HTT does not increase genomic mutation rate during continuous culture of PSC in comparison to control cells. Therefore, HD cells should be assessed for mutations as frequently as control PSC. In addition, the substantial expansions characteristic of in vivo conditions may not be identified prior to months of culture of differentiated neurons. The application of stress to cells may enhance CAG and genomic instability.

## Developmental Hallmarks of Disease in Stem Cell Modeling HD

### HTT Expression and Aggregation

HTT is an ubiquitously expressed protein [113]; however, its level increases along with brain development [114] or following the in vitro differentiation of PSC into neural lineages [48, 85]. Moreover, multiple non-canonical HTT isoforms that result from alternative splicing have been described in normal human cells and HD ESC [115]. One alternatively spliced transcript, which excludes HTT posttranslational cleavage-regulating exon 10, has been downregulated during neuronal differentiation, which suggests a role in development. Of the 3 isoforms previously identified in HD patients and mice [116–118], none has been identified in ESC [115].

Mutant HTT forms various oligomeric and polymeric aggregates that differ in terms of protein composition, solubility, cellular localization, and toxicity [119, 120]. The presence of antibody-detectable inclusions in cells of the CNS and peripheral tissues is a hallmark of disease. However, they are absent in immature PSC and NSC and only begin to emerge in mouse [22] and rhesus [39] transgenic HTT-exon 1 (144Q and 65-72Q, respectively) cells after 1.5–3 weeks of neural differentiation. The formation of inclusions is preceded by the formation of soluble oligomers in rosette-stage NSC, which may be detected via immunoblotting. In human transgenic ESCs with mutant HTT, an exon 1 fragment with juvenile CAG repeat range inclusions and soluble oligomers were identified approximately 2 months after differentiation [37]. However, a longer time span is required for the formation of detectable inclusions in juvenile and adult patient-derived cells because inclusions were detected in neurons after 6 months of culture [25] or transplantation [38], but not up to 2–3 months of differentiation [26, 33]. Aggregation may be enhanced with the use of a proteasome inhibitor, e.g., MG132. In one study, the use of MG132 enabled researchers to identify inclusions already in human iPSC with 72Q [38]. Interestingly, mutant HTT RNA also aggregates into toxic foci, which may be identified in human iPSC and derived NPC with only 57 CAG repeats [97, 98]. Observations in human cells reveal the late onset of aggregation identified in HD patients and lack of aggregates in early developmental stages of cellular HD models.

### Differences in Gene Expression

Gene expression alterations may be the most common and earliest difference detectable in HD cells at the PSC or NSC stage. We have reviewed the research regarding alterations in gene expression and performed a simple meta-analysis to retrieve level 5 GO terms (most detailed GO level) related to developmental biology and signaling pathways related to developmental processes using lists of names of deregulated genes provided by 8 research works. The “Methods” section contains detailed meta-analysis paradigm, and Table 2 contains a list of research works that were included in the analysis.

#### Methods

**Fig. 3:**
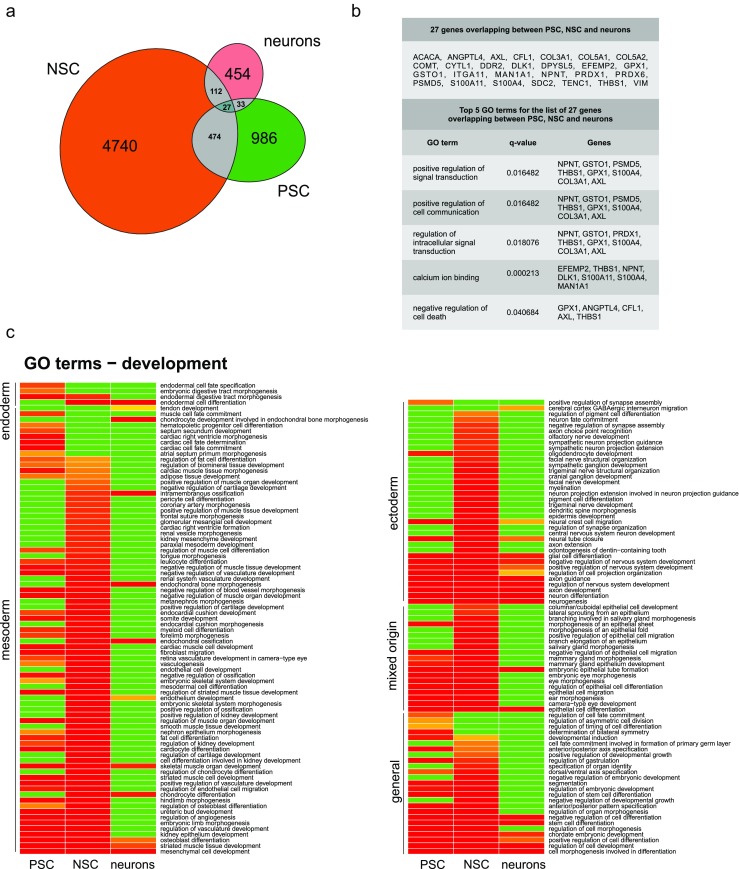
Meta-analysis reveals common genes/proteins and developmental GO terms for PSC, NSC, and neurons.**a** Names of genes that overlap between the lists of deregulated genes for PSC, NSC, and neurons. Venn diagram indicates number of genes/proteins included in the meta-analysis that overlap between only two or all three of the analyzed lists of genes. Detailed list of overlapping genes is available in Supplementary Fig. 2 (Venn diagram generated with eulerAPE v3). **b** *Top*: list of 27 genes/proteins that overlap between all three lists of genes for PSC, NSC, and neurons. *Bottom*: top 5 GO terms (level 5) containing the highest number of genes/proteins from the input list with 27 genes. The *q* values and list of members are provided. **c** Heatmap demonstrates GO terms (level 5) related to development, which were detected by the meta-analysis in the lists of genes for PSC, NSC, and neurons. Absence of developmental GO term (*green*), the *increasing dose* of *warm colors* (*orange* to *red*) denotes the increasing statistical significance of GO term (decreasing *q* value for a particular term). GO terms in the heatmap are ordered according to germ layers and general events (Heatmap generated with R software v3.3.0) (Color figure online)

Eight works listed in Table 2, all investigating human cells, were selected for the meta-analysis. The names of the human deregulated genes or proteins represented as names of genes were retrieved from 8 original works and were sorted into 3 separate lists. Names of genes and names of genes corresponding to deregulated proteins were subsequently listed as HUGO Gene Nomenclature Comity symbols (HGNC symbol). We established a list containing names of genes deregulated in NSC and iPSC, a list containing names of genes deregulated in NSC, both ESC and iPSC derived, and a third list containing names of genes deregulated in neurons. We did not distinguish between the deregulated genes/proteins identified in transcriptomic and proteomic experiments, since the aim of the meta-analysis was to identify level 5 GO terms and overrepresented signaling pathways related to developmental biology to generate a global overview reflecting consequences of deregulations of genes and proteins for the cellular phenotype. The cell types, culture conditions, and high-throughput methods used by the authors to identify the deregulations, together with the cutoff values selected in the 8 studies are indicated in Table 2 and Suppl. Table 1. The genes overlapping between lists, and the genes reported in more than one of the 8 studies included in the meta-analysis, were identified using MS Excel formulas (Fig. 3a, b, Suppl. Table 2).

**Table 2:** Studies included in the meta-analysis

Cell type	Markers of cell identity	Method	Cutoff	Reference
iPSC and iPSC-derived NSC	Nestin (NSC)	RNA-seq	FDR <0.05	[42]
ESC and ESC-derived NSC	Nestin, PAX6, Ki67 (NSC)	Gene microarray	*P* < 0.001	[76]
iPSC	Markers for pluripotency	Gene microarray	FDR <0.05	[24]
iPSC-derived NSC and neurons	Nestin, PAX6 (NSC) TUBB3,MAP2A/B, DARPP-32, BCL11B (neurons differentiated towards striatal-like)	Gene microarray	absolute value of fold change >2	[26]
iPSC-derived neurons	TUJ1, MAP2, GABA, GAD65, DARPP-32, Calbindin (neurons differentiated towards GABAergic striatal like)	Gene microarray	Adjusted *P* < 0.05 and fold change >1	[33]
iPSC-derived neurons	TUBB3, DARPP-32, GAT1 (neurons differentiated towards GABAergic MS-like)	Gene microarray	*P* < 0.05	[25]
ESC and ESC-derived neurons	MAP2, GABA, GAD65 (neurons differentiated towards GABAergic-like)	IPG-IEF and LC-MS/MS	*P* < 0.05	[34]
iPSC and iPSC-derived neurons	MAP2 (neurons)	Western blot, 2D electrophoresis and LC-MS/MS	*P* < 0.05	[47]

For the retrieval of level 5 GO terms, each list was separately subjected to overrepresentation analysis using the respective tool included in an online version of ConsensusPathDB (CPDB) [121]. “Biological process,” “molecular function,” and “cellular component” GO terms (level 5, *p* value cutoff = 0.01) have been selected for the analysis. The retrieved GO terms for each gene list were further manually sorted into 22 arbitrary categories (Suppl. Table 4). The GO annotations related to development were presented in the form of a heatmap (Fig. 3c) [122, 123] using the q values (adjusted p values) calculated with the use of overrepresentation analysis in CPDB. The green heatmap color denoted the lack of GO term, and the increasing dose of warm colors denoted the presence and increasing statistical significance of GO terms (decreasing q value). For retrieval of biological pathways, each list of genes was again separately subjected to overrepresentation analysis using the respective tool of CPDB, and statistically significant pathways were retrieved using the *p* value cutoff = 0.01, and minimum overlap with the input list equaled 10 genes (Suppl. Table 5).

#### GO Terms Identified for Deregulated Genes

The list of names of differentially expressed genes/proteins, used for meta-analysis, contained 986, 4740, and 454 names of genes for PSC (ESC and iPSC), NSC, and neurons, respectively. Among the listed genes, several deregulated genes overlapped between the analyzed lists; however, only 27 genes were present in all 3 lists (Fig. 3a, b, Suppl. Table 2). Supplementary Tables 2, 3, 4, and 5 present the detailed results of our meta-analysis, including the genes/proteins overlapping between the lists, all GO terms, and genes/proteins reported in more than one study and biological pathways that were represented by more than ten genes. As also described in the “Methods” section, we have established a total number of 22 arbitrary categories. We have selected ten arbitrary categories which represented biological processes related to the developmental aspect of HD and categories with already well-established HD phenotypes. Table 3 indicates the selected categories, with the number of related GO terms and the top 5 GO terms in a particular category. The top 5 GO terms were selected by the highest number of genes/proteins from the input list. In addition, the number of GO terms in a particular category was normalized to the total number of GO terms identified for a list of genes of a particular cell type and also presented as the percentage of total number of GO terms (Table 3). The same or similar top 5 GO terms have been shared for 2 or 3 lists of genes in several of the arbitrary categories. The categories sharing GO terms between all gene lists included “differentiation,” “neurodevelopment,” “cytoskeletal organization, cell-cell interactions,” and “apoptosis”. The categories sharing GO terms between two gene lists included “proliferation” (NSC and neurons), “development,” “control of gene expression,” and “metabolism” (PSC and NSC). These findings suggest that similar biological processes, resulting from deregulation of genes/proteins, occur in PSCs and may be sustained during differentiation, until reaching the stage of mature neurons. Noteworthy, similar top 5 GO terms occurred in the analysis, regardless of the protocols for ESC/iPSC derivation, NSC differentiation, neuronal identity, and the inclusion of genes from transcriptomic and proteomic studies. This analysis also confirms that the investigation of HD-associated changes, along the differentiation axis of ESC/iPSC-NSC-neurons, is justified. Finally, the categories most strongly represented by GO terms in PSC and NSC namely, differentiation, neurodevelopment, and development highlight the role of developmental processes, resulting from gene deregulations in HD.

**Table 3:** Meta-analysis summary: number of GO terms and names of top 5 GO terms grouped into 10 arbitrary categories describing relevant biological and developmental processes and HD phenotypes. Each arbitrary category contains the indicated number of GO terms, which is also expressed as a percentage of total number of GO terms for particular cell type. Numbers in brackets indicate number of genes from the input list, which were assigned by CPDB for each GO term

Category	PSC—292 total GO terms, 986 genes	NSC—445 GO terms, 4740 genes	Neurons—191 GO terms, 454 genes
Proliferation, cell cycle, and growth	13 GO terms; 4.45 %	21 GO terms; 4.80 %	10 GO terms; 5.23 %
Top 5 terms	Positive regulation of cell proliferation (62), negative regulation of cell proliferation (50), regulation of mitotic cell cycle (32), regulation of epithelial cell proliferation (31), positive regulation of cell cycle (21)	Positive regulation of cell proliferation (276), negative regulation of cell proliferation (224), regulation of cell cycle process (146), negative regulation of cell cycle (139), mitotic cell cycle phase transition (130)	Positive regulation of cell proliferation (34), negative regulation of cell proliferation (27), regulation of cell cycle process (20), negative regulation of cell cycle (19), positive regulation of cell cycle (16)
Differentiation	24 GO terms; 8.22 %	24 GO terms; 5.39 %	9 GO terms; 4.71 %
Top 5 terms	Cell morphogenesis involved in differentiation (89), positive regulation of cell differentiation (67), negative regulation of cell differentiation (53), epithelial cell differentiation (45), stem cell differentiation (42)	Cell morphogenesis involved in differentiation (378), positive regulation of cell differentiation (297), negative regulation of cell differentiation (221), epithelial cell differentiation (204), stem cell differentiation (151)	Cell morphogenesis involved in differentiation (58), negative regulation of cell differentiation (30), positive regulation of cell differentiation (29), epithelial cell differentiation (27), stem cell differentiation (19)
Development	59 GO terms; 20.21 %	93 GO terms; 20.90 %	12 GO terms; 6.28 %
Top 5 terms	Regulation of cell development (61), chordate embryonic development (58), regulation of cell morphogenesis (43), striated muscle tissue development (38), regulation of vasculature development (30)	Regulation of cell development (303), chordate embryonic development (243), regulation of cell morphogenesis (193), striated muscle tissue development (138), camera-type eye development (117)	Regulation of cell development (35), chordate embryonic development (25), striated muscle tissue development (16), endochondral bone morphogenesis (12), mesenchymal cell development (12)
Neurodevelopment	13 GO terms; 4.45 %	30 GO terms; 6.74 %	12 GO terms; 6.28 %
Top 5 terms	Neurogenesis (121), neuron differentiation (109), axon development (58), Regulation of nervous system development (54), axon guidance (44)	Neurogenesis (578), neuron differentiation (504), regulation of nervous system development (282), axon development (264), axon guidance (184)	Neurogenesis (74), neuron differentiation (60), axon development (36), regulation of nervous system development (30), axon guidance (27)
Control of gene expression	18 GO terms; 6.16 %	16 GO terms; 3.60 %	4 GO terms; 2.09 %
Top 5 terms	Regulation of gene expression (206), transcription, DNA-templated (179), sequence-specific DNA binding (63), regulatory region DNA binding (52), transcriptional activator activity, RNA polymerase II core promoter proximal region sequence-specific binding (21)	Regulation of gene expression (1057), transcription, DNA-templated (901), sequence-specific DNA binding (281), regulatory region DNA binding (240), transcriptional activator activity, RNA polymerase II core promoter proximal region sequence-specific binding (91)	Structure-specific DNA binding (16), DNA conformation change (12), protein-DNA complex assembly (10), establishment of protein localization to chromosome (2)
Signaling pathways	18 GO terms; 6.16 %	29 GO terms; 6.52 %	8 GO terms; 4.19 %
Top 5 terms	Cell surface receptor signaling pathway (166), intracellular signal transduction (151), positive regulation of signal transduction (94), regulation of intracellular signal transduction (90), negative regulation of signal transduction (71)	Cell surface receptor signaling pathway (839), intracellular signal transduction (805), regulation of intracellular signal transduction (454), positive regulation of signal transduction (430), negative regulation of signal transduction (325)	Cell surface receptor signaling pathway (90), intracellular signal transduction (82), regulation of intracellular signal transduction (50), positive regulation of signal transduction (42), negative regulation of signal transduction (37)
Cytoskeletal organization, cell-cell interactions	13 GO terms; 4.45 %	22 GO terms; 4.94 %	14 GO terms; 7.33 %
Top 5 terms	Positive regulation of cell communication (106), negative regulation of cell communication (75), actin cytoskeleton (33), positive regulation of cell adhesion (25), regulation of cell-cell adhesion (24)	Positive regulation of cell communication (506), microtubule cytoskeleton (274), negative regulation of cell communication (355), actin cytoskeleton (182), actin filament organization (117)	Positive regulation of cell communication (52), negative regulation of cell communication (38), actin cytoskeleton (29), positive regulation of cell adhesion (20), actin filament organization (18)
Oxidative and other cell stress	3 GO terms; 1.03 %	11 GO terms; 2.47 %	10 GO terms; 5.24 %
Top 5 terms	Cellular response to reactive oxygen species (12), stress fiber (8), response to X-ray (5)	Stress-activated MAPK cascade (82), response to UV (43), cellular response to reactive oxygen species (40), cellular response to alcohol (37), stress fiber assembly (32)	Cellular response to reactive oxygen species (11), stress fiber (7), regulation of oxidative stress-induced cell death (6), cellular response to ionizing radiation (6), regulation of response to oxidative stress (6)
Apoptosis	6 GO terms; 2.05 %	8 GO terms; 1.80 %	10 GO terms; 5.24 %
Top 5 terms	Apoptotic process (112), regulation of programmed cell death (97), negative regulation of cell death (67), positive regulation of cell death (40), neuron apoptotic process (24)	Apoptotic process (570), regulation of programmed cell death (447), negative regulation of cell death (289), apoptotic signaling pathway (198), positive regulation of cell death (197)	Apoptotic process (73), regulation of programmed cell death (66), negative regulation of cell death(48), apoptotic signaling pathway (27), positive regulation of cell death (26)
Metabolism	42 GO terms; 14.38 %	61 GO terms; 13.71 %	33 GO terms; 17.28 %
Top 5 terms	Regulation of cellular biosynthetic process (203), regulation of nucleobase-containing compound metabolic process (198), regulation of macromolecule biosynthetic process (187), positive regulation of cellular metabolic process (183), RNA biosynthetic process (182)	Regulation of cellular biosynthetic process (1055), regulation of nucleobase-containing compound metabolic process (999), regulation of macromolecule biosynthetic process (996), RNA biosynthetic process (926), regulation of RNA metabolic process (907)	Positive regulation of cellular metabolic process (87), positive regulation of macromolecule metabolic process (76), regulation of protein metabolic process (75), regulation of cellular protein metabolic process (68), negative regulation of cellular metabolic process (65)

Nevertheless, there are also differences between the analyzed lists of genes. As expected, there were fewer GO terms related to development for neurons than for PSC or NSC. A plausible explanation is the fact that the genes and processes that play a role in the developmental process may have biological functions in mature neurons. There were twice as many GO terms in the neurodevelopment category for NSC than for other lists of genes, which reflects the developmental stages of the analyzed cells. Another evident difference was identified in the metabolism category. The GO terms related to metabolism of nucleic acids dominate among the deregulated genes listed for PSC and NSC, which can likely be attributed to high proliferation rates, whereas in neurons, the category contains mainly GO terms related to metabolism of proteins and neurotransmitters. Furthermore, there is no GO term related to lipid metabolism in neurons, whereas it is present in the top 5 GO terms in the “cell response” category for PSC and NSC (Suppl. Table 4), which may be associated with a higher demand for cholesterol in the early stages of embryonic development [22]. Interestingly, NSC and neurons are enriched in GO terms related to cell stress compared to fewer GO terms in PSC. This finding corresponds with the demonstration revealing that after differentiation, HD cells are more vulnerable to stress and death evoked by withdrawal of BDNF and other growth factors [24]. There have been several, additional interesting categories and GO terms representing biological processes, implying their role in HD. For instance, in the meta-analysis, there have been such GO terms as “response to wounding” or “axon regeneration” (Suppl. Tables 3 and 4), which emphasizes the occurrence of processes related to neural degeneration in HD or GO terms related to immune response underlining its important role in HD [124]. In our analysis, we did not detect GO terms directly related to epigenetics. However, these changes were detected in mouse and human ESC, NPC, and neurons that linked mutant HTT and chromatin status during development [32, 34].

### Signaling Pathways

Several signaling pathways involved in embryonic development are also affected in HD [125]. For example, the MEK/ERK signaling pathway, which is a part of the MAPK signaling pathway, plays a neuroprotective role in HD. In addition, the TGF-beta signaling pathway is upregulated in HD human cells and a rat model [125]. The meta-analysis performed for the purposes of this review also identified overrepresented, developmental signaling pathways among the deregulated genes and proteins identified in iPSC and NSC, collected from the eight research works and listed in Table 2. In iPSC, the developmental signaling pathways [126], represented by at least ten deregulated members (proteins/genes) as a cutoff (cutoff = 10, *p* < 0.01), included the TGF-beta, Wnt, PI3K-Akt, Hippo, MAPK, EGFR1, and Rap1 signaling pathways semaphorin interactions, and other signaling pathways. In NSC, the pathways included TGF-beta, beta catenin, PI3K-Akt, EGFR1, PDGFR-beta, Rap1, Hippo, BDNF, semaphorin interactions, and other pathways (cutoff = 10, *p* < 0.01). In terminally differentiated neurons, the signaling pathways with at least ten deregulated proteins included the MAPK family, PI3K-Akt, EGFR1, PDGF, NGF, and other (cutoff = 10, p<0.01) (Suppl. Table 5). In addition to high-throughput screens, the HD signaling pathways have also been investigated in a more detailed way, and early HD phenotypes have been identified in stem cells. For instance, the deregulation of the MAPK, Wnt, and p53 pathways was identified in mouse and human iPSC [48]. A study by Ring et al. indicates that NSC derived from HD human iPSC (72Q) were rescued from apoptosis when TGF-beta was added to growth factor-deprived culture medium (without bFGF and LIF) [42]. However, the apoptotic phenotype was masked in a complete culture medium, potentially because the TGF-beta signaling, which is putatively protective, was upregulated in the HD NSC. Therefore, it must be noted that the signaling pathway inhibitors and activators used during stem cell culture and differentiation may modify or mask the HD phenotypes related to their activity in vivo.

The p53 tumor suppressor protein is a regulator of cell fate specification, as the p53-null mutation in mice leads to multiple developmental defects, including brain exencephaly [127, 128]. Embryonic lethality may be induced by disrupting p53 transactivation functions via the KO of the p53 regulators, the E3 ubiquitin-protein ligases: MDM2 or MDM4 [129], or the transcription activation domains, vital for interactions [130]. In HD, p53 influences the disease phenotype via multiple pathways, which have previously been reviewed in a comprehensive way [125]. In addition, the absence of p53 in Hdh^140Q/140Q^ mouse, which presents a number of repeats in the juvenile range, results in increased formation of aggregates in the brain [131]. Research on adult HD cells or HD patient brains indicates that the p53 expression or activity was upregulated at later stages of disease and mediated apoptosis [125].

In human undifferentiated and neuronally differentiated HD iPSC (86Q), p53 was extensively translocated into mitochondria, which was accompanied by p53-dependent activation of pathways that led to neuronal maturation defects and apoptosis [43]. Both defects were repaired by p53 silencing or mitochondria fission inhibition [43]. Moreover, Chae et al. identified increased levels of p53 phosphorylation in human HD iPSC (71 CAG) [47]. In contrast, we have shown that p53 is downregulated in undifferentiated mouse iPSC from the YAC128 model (128 CAG) and also in human iPSC sampled from a juvenile HD patient (onset at 3 years; 109 CAG) [43]. The p53 remained unchanged in iPSCs independently derived from the same patient as in the work of Chae et al. containing 71 CAG repeats (disease onset at 14 years) [48]. Moreover, p53 has been identified as a top 5 gene regulator of the pathways altered after HTT mutation correction in hiPSC-derived NSC [42]. It is plausible that the p53 deregulation profile in juvenile HD stem cells is dependent on the number of CAG repeats. It is also possible that the expression level and activity of p53 may be different in juvenile HD and in adult HD. However, to verify these hypotheses, data from more HD iPSC lines, preferably isogenic and ranged from low to high CAG repeat numbers, are needed. The mitochondrial and apoptotic effects that may result from altered p53 activity will be discussed in subsequent sections.

### Mitochondrial Dysfunction

One of the most extensively investigated features of HD pathology includes complex mitochondrial impairments involving multiple aspects of organelle biology [132–134]. However, their genesis and impact on disease pathogenesis are insufficiently understood. The downstream consequences of these changes may include susceptibility to excitotoxicity, reactive oxygen species (ROS)-induced DNA damage, and apoptosis.

The mitochondrial content, life cycle, activity, and dynamics are important regulators of embryonic and brain development, and their dysfunction may lead to neurodevelopmental disorders [135]. Remarkably, Ismailoglou et al. reported that mouse ESC with HTT KO had increased glucose uptake from the medium. The cells were incapable of sufficient mitochondrial ATP synthesis and therefore turned to glycolytic respiration and lowered their oxygen consumption [41]. The mitochondria in these cells were aberrantly structured; however, no polarization defects were identified. Similarly, the ESC containing mutant HTT(140Q) from knock-in HD model exhibited increased glucose consumption; however, in contrast to KO cells, the oxidative phosphorylation respiration increased, with no evidence of alterations in the mitochondrial structure. On the other hand, Jacobsen et al. reported no ATP/ADP ratio changes in KO mouse ESC and a decreased ATP/ADP ratio in Q111 knock-in cells [40]. The discrepancy between these two studies likely resulted from the different culture conditions and different reference cells. Ismailoglou et al. cultured cells in feeder-free and defined serum-free ground-state (2i+LIF) conditions [136], whereas Jacobsen et al. maintained the cells in undefined serum replacement conditions on feeders, with LIF only. Nevertheless, these studies, combined with proteomic research on human ESC and iPSC [34, 47], have identified a substantial number of alterations in the metabolome and expression of genes related to mitochondrial function, energy metabolism, and metabolite synthesis. The metabolic impairments induced by the mutation or loss of the *HTT* gene include dysregulation of the lipid and cholesterol synthesis pathways, which have previously been shown to be affected in patients [137], PSC [22, 34, 41], and differentiated neurons [26].

Research regarding human iPSC-derived NSC has indicated that calcium signaling and the ATP/ADP ratio are decreased in these cells [26], and genetic correction of mutant HTT alleles enhanced the maximum respiration rate towards control cell levels [24]. Guo et al. focused on mitochondrial fission deficits in multiple cellular and mouse models of HD [43]. In their models, excessive accumulation of the fission-driving protein Drp1 causes the translocation of p53 to mitochondria, which leads to accumulation of ROS, mitochondrial fragmentation, and, consequently, apoptosis. In HD iPSC, they identified increased levels of Drp1 and p53 in mitochondria, whereas iPSC-derived GABAergic neurons, including MSNs, had defective, fragmented mitochondria neurites with a decreased membrane potential, decreased ATP/ADP ratios, increased ROS, and enhanced apoptosis. Both the selective Drp1 inhibitor P110-TAT and p53 silencing rescued these phenotypes and normalized the neurite lengths. The treatment also turned out to be beneficial in R6/2 mice, which validated research in iPSC HD cellular models.

The altered ROS accumulation and oxidative stress response represent other mitochondria-related impairments featured in HD [138]. As described, an increased ROS content has been identified in human MSNs [43]. If not neutralized, excess ROS may induce DNA damage and alter ROS signaling pathways, which may regulate proliferation and differentiation processes [139]. The phenotype was also shown in mouse knock-in ESC- and brain-derived NSC with 140Q [28]. Interestingly, the loss of HTT in KO-NSC did not cause ROS accumulation. In human iPSC, the expression levels of multiple antioxidant proteins were altered, including the downregulation of SOD1 and GST and the upregulation of proteins in the PRX family [47]. These changes were sustained throughout differentiation into mature neurons. We have also shown [48], in both adult and juvenile human iPSC and mouse YAC128 iPSC, that SOD1 is altered; however, in our case, it was upregulated. This discrepancy again points to the potential effects of the culture conditions, as we used feeder-free defined Essential 8 conditions for culture of human iPSC. Nevertheless, these changes may indicate that an altered redox homeostasis and increased susceptibility to oxidative stress, including the ultimate solution, apoptosis (which will be described later), are present in iPSC.

Another crucial mitochondria-related pathomechanism of HD is excitotoxicity, in which *N*-methyl-d-aspartate receptors (NMDARs) are overactivated in response to glutamate. NMDAR overstimulation leads to dysregulation of the cellular Ca^2+^ homeostasis, which is typically maintained by mitochondria and endoplasmic reticulum, and ultimately leads to cell death [140, 141]. The propensity for neuronal excitotoxicity in mature brains has been evaluated using ESC/iPSC-derived neurons. The calcium homeostasis was disrupted in MSN-like cells derived from human HD iPSC [25]. The abnormal increase in the cellular calcium entry, which was regulated by calcium store-operated channels, was effectively blocked by EVP4593, thereby leading to decreased cell death rates. Exposure to both physiological and pathological glutamate concentrations exacerbated the Ca^2+^ imbalance and led to increased apoptosis in striatal-like HD cell lines, from adult and juvenile patient iPSC [26]. It has subsequently been demonstrated that the cells that died in this in vitro culture were not mature neurons; instead, they were Nestin+ striatal-like NPC, and the excitotoxicity may be mediated by the loss of neuroprotective BDNF via the TrkB pathway [30]. BDNF is a key player in HD pathogenesis [141], and its downregulation has been identified following differentiation to NSC [24, 44].

It is still to be determined how mitochondrial deficits correspond to defects in the differentiation and maturation of HD cells. Moreover, the early presence of a mitochondrial phenotype in pluripotent models may provide additional insights into the mitochondrial impairments in HD, which are of particular interest as a result of several contradictory observations concerning metabolism and mitochondria in HD [132].

### Autophagy and UPS

Autophagy is a part of the stress response system responsible for the degradation of dysfunctional or toxic protein aggregates and organelles, including mitochondria, and pathogens. In this process, proteins and organelles to be cleared are delivered to lysosomes, the effectors of degradation. Autophagy is important for stem cell self-renewal and development, and it is essential for the maintenance of stress-sensitive, postmitotic neurons [142, 143]. In HD, mutant HTT impairs the autophagosome trafficking and thus the fusion of autophagosomes with lysosomes [144]. The mRNA expression of *Tfeb*, a master regulator of autophagy and lysosomes, along with their targets, *Tpp1* and *Ctsf*, has been shown to be increased in iPSC and neurons derived from R6/2 mice [22]. These increases resulted in a greater number of lysosomes in iPSC. An increased number of lysosomes have also been identified in an adult patient’s iPSC and the derived neurons [23]. Additional studies have confirmed an increased number of autophagosomes, lysosomes, and mitophagy (mitochondrial autophagy) in iPSC-derived MSN-like neurons [25]. In these cells, lysosome content was decreased following treatment with the Ca^2+^ influx-repairing agent EVP4593. Interestingly, astrocytes derived from the juvenile HD patient iPSC had an increased content of cytoplasmic vacuoles, some of which were autophagosomes [94].

Another mechanism for clearance of unwanted proteins is the unfolded protein response (UPR)/ubiquitin-proteasome system (UPS) pathway. In HD, the accumulation and aggregation of mutant HTT result from a failure of the UPS to efficiently deal with polyQ-expanded HTT [144, 145], which may be influenced by deficits in the preceding UPR pathway [146]. Consistent with this evidence, human ESC and derived neurons demonstrate altered levels of the ubiquitination pathway proteins [34], whereas correction of the HD mutation in human iPSC results in the upregulation of UPR-related gene expression [24, 146].

### Apoptosis

Programmed cell death may be mediated by apoptosis, which is activated by a caspase cascade. Caspase activity is elevated in cellular and animal models of HD and postmortem brain tissues obtained from HD patients [147]. In vivo experiments have also shown that modulation of the HTT level results in apoptotic responses in the developing brain. For instance, the depletion of HTT in neuroepithelial cells by the shRNA construct may lead to disturbed cell migration from ventricular zone to caudoputamen and reduced proliferation or increased apoptosis in the cortex of developing 12.5-day-old embryo. Both the neuronal survival and proliferation may be partially rescued by over 40% caspase-9 knockdown [148]. Notably, mutant Hdh CAG knock-in NSC exhibit overactivity of caspase-3/7 [147, 149]. Similarly, the activity of caspase-3/7 plays a key role in apoptosis in HD, and it is increased in a CAG repeat length-dependent manner in mouse ESC-derived NSC during differentiation [27]. Even the cells without endogenous HTT exhibit high levels of caspase activity [150, 151]. However, mutation of the *HTT* gene in human ESC or iPSC did not induce programmed cell death, as indicated by the activated caspase-3 staining [23]. Furthermore, there was no difference in the caspase-3/7 activity between genetically corrected (21 CAG repeats) and uncorrected human isogenic HD iPSC (72 CAG repeats) [24]. Both corrected and uncorrected HD iPSC were differentiated into NSC, followed by stress induction through withdrawal of growth-supporting factor from the culture medium. The uncorrected HD NSC responded to such a withdrawal with increased apoptosis as assessed by increased TUNEL staining and increased caspase-3/7 activity, while the corrected NSC were “cured” of these effects. The results suggest that polyglutamine expansion makes iPSC and NSC more sensitive to cell death on their differentiation route towards neurons; however, they become sensitive to polyQ-induced apoptosis at a relatively early stage of neuronal development.

Apoptosis was also increased following stress conditions in cells obtained from HD patients or animal models [147, 152, 153]. In the pluripotent HD models, after 3 weeks of differentiation of juvenile human NSC, there has been a gradual decrease in the number of active neurons, with eventual cell death by the end of the third week [26]. The HD cultures developed a severe phenotype and exhibited high levels of mutant HTT expression. Furthermore, after differentiation using a modified, more protective protocol, neurons exhibited increased caspase-3/7 activity and mortality following BDNF withdrawal. This effect was reversed by the addition of BDNF, at 4 times of its normal concentration, to the media of the HD cell cultures [26]. The selective inhibition of ATM-mediated signaling may also confer the protection of HD iPSC-derived striatal neurons from BDNF withdrawal [45]. Multiple reports have identified stressors that induce increased levels of apoptosis in PSC-derived HD neurons, e.g., H_2_O_2_ (oxidative stress) [33, 39], 3-methyladenine (autophagy inhibitor), MG-132 (proteasome inhibitor) [105] and staurosporine (broad-spectrum kinase inhibitor) [34]. In HD, neuronal dysfunction and death are more widespread, notably in the cases of longer CAG repeats, which suggests that the toxicity mediated by stress factors may not be exclusively limited to striatal neurons. It should be noted that severe MSN death characteristic for HD is not as relevant in mouse models. For example, in knock-in mice, the phenotype can hardly be observed until late life unless mice are homozygous and have CAG repeat numbers over >140. Therefore, human and primate HD PSC are more suitable for research on apoptosis.

### Cell Adhesion

Cell adhesion and cytoskeletal molecules, including N-cadherin and actins, are indispensable for normal brain development because of their vital roles in cell orientation, migration, communication (including apoptotic signaling), and the formation of brain structures [154, 155]. As previously discussed, in the absence of HTT, the rosetteless phenotype may occur in NSC as a result of increased activity of ADAM10 metalloproteinase, which mediates increased cleavage of N-cadherin [65]. Moreover, N-cadherin deficits have been identified in the brains of HD Q111 knock-in mice [156].

The N-cadherin pathway is directly affected by mutant HTT, and genetic correction of the mutant allele has resulted in upregulated expression of several protocadherins in human iPSC and iPSC-derived NSC [24, 42]. Moreover, human iPSC-derived NSC lines with 60Q and 180Q bound less phalloidin peptide, which suggests changes in the actin cytoskeleton, and displayed a decrease in the adhesive capabilities via cell cluster formation assays [26]. The existence of actin deficits in HD NSC is further supported by the motility reduction identified in mouse ESC-derived NSC, with a 110Q knock-in mutation or the loss of HTT [28], as well as the expression changes in the motility and cytoskeleton pathways, identified following neuronal differentiation of 140Q knock-in mouse ESC, human ESC, and iPSC [19, 26, 34, 47]. Taken together, these findings suggest that cytoskeletal and adhesion molecules may influence the functional differentiation and survival deficits that occur in stem cell models of HD and during in vivo development of HD models.

## Conclusion

In conclusion, this review summarizes current evidence for the existence of a developmental, pathogenic phase in the progression of HD, which suggests that the traditional view that HD is solely an adult, neurodegenerative disease should be revisited. The most convincing evidence includes the effects of induced expression and the selective deficiency of normal or mutant HTT in developmental stages. Potential mechanisms are presented, based on data from in vivo models. Pluripotent, neuronal stem cells and mature neurons derived from these cells also exhibit an array of phenotypes characteristic of HD patients and models. Finally, the GO terms retrieved for the deregulated genes in HD cell models and deregulated signaling pathways (most importantly, TGF-β, Wnt, and MAPK signaling) are directly related to development and neurogenesis, affected in vitro and in vivo. Taken together, ESC/iPSC appear to be the best cellular models, which are available at present to investigate the impact of neurodevelopmental defects in HD. The data also indicate the need for optimization of human iPSC models, including reprogramming and differentiation protocols, the use of isogenic human stem cells, cerebral organoids, and defined media, for the purposes of consistent observation of HD phenotypes. The selective differentiation of ESC/iPSC into neuronal cell types provides an additional opportunity to model late HD phenotypes because the neurons may be maintained for extended periods of time in culture. In this model, later phenotypes may be enhanced by the application of stressors or aging factors that induce age-related events, such as progerin [157, 158]. The developmental characteristics of HD have crucial implications for therapy. It remains unclear which therapeutic strategy is most appropriate and when the treatment should be initiated. For cell therapy, a transplant may directly provide protective and trophic agents, or it may be designed to mature in vivo, developing functional neurons within the network of host cells.

Neurodegenerative disorders are traditionally recognized as diseases of late onset; however, this perception may be shifting. A suitable example demonstrating the need for this perception change can be the evidence showing that healthy 3-year-olds in a genetic risk group of developing Alzheimer disease achieved lower scores on working memory and attention and also had smaller hippocampi than the non-risk group representatives [159].

CNS, central nervous system; DG, dentate gyrus; ESC, embryonic pluripotent stem cell; GO, Gene Ontology; HD, Huntington disease; HTT, huntingtin; iPSC, induced pluripotent stem cells; KO, knockout; MSN, medium spiny neuron; NPC, neural progenitor; NSC, neural stem cells; polyQ, polyglutamine; PSC, pluripotent stem cells; SVZ, subventricular zone; WT, wild type

## Electronic Supplementary Material

Supplementary Table 1. The table contains cell source, cell culture conditions and differentiation conditions used in the studies included in the meta-analysis. (DOCX 27 kb)

Supplementary Table 2. The table contains 3 separate lists of deregulated genes for PSC, NSC and neurons which were used in Consensus Pathway Data Base to retrieve GO terms and signaling pathways. The table also contains lists of genes overlapping between genes reported in more than one high throughput study. (XLSX 96 kb)

Supplementary Table 3. List of GO terms (level 5) retrieved in the meta-analysis for each of the lists of genes deregulated in PSC, NSC and neurons. (XLSX 400 kb)

Supplementary Table 4. List of all 22 arbitrary categories containing GO terms (level 5) retrieved in the meta-analysis for PSC, NSC and neurons. (XLSX 17 kb)

Supplementary table 5. List of signaling pathways retrieved in the meta-analysis for PSC, NSC and neurons. (XLSX 130 kb)
