# Supplementary material for: Huntington Disease as a Neurodevelopmental Disorder and Early Signs of the Disease in Stem Cells
Source: Mol Neurobiol. 2017 May 11;55(4):3351–71. doi: 10.1007/s12035-017-0477-7 (PMC5842500; doi:10.1007/s12035-017-0477-7)
Supplement: Supplementary file 1 — The table contains cell source, cell culture conditions and differentiation conditions used in the studies included in the meta-analysis. (DOCX 27 kb) [file 12035_2017_477_MOESM1_ESM.docx]

Suppl. Table 1

| **Cell type** | **Cell lines *** | **Passage **** | **Plate coating and culture medium **** | **Ref.** |
| --- | --- | --- | --- | --- |
| iPSC | Isogenic lines  HD: HD iPSC described in [24],  Control line: genetically corrected C116 iPSC (21 CAG) described in [24],  Non-isogenic iPSC lines (Coriell):  ND41656 (57 CAG),  ND42222 (109 CAG) | 1 mg/ml collagenase | Cultivation  Matrigel (BD Biosciences);  medium: mTeSR1 defined medium (Stemcell) | [42] |
| NSC | Derived from iPSC [42] | Accutase (Sigma) | Differentiation  medium: STEMdiff Neural Induction Medium (Stemcell) with 10 μM Y27632 ROCK inhibitor (Calbiochem). After 7 days addition of STEMdiff Neural Rosette Selection Reagent | [42] |
|  |  |  | Cultivation  poly-L-ornithine/ laminin, Matrigel (Corning);  medium: Neurobasal medium with 1XB27,  2mM L-Glutamine,  25ng/ml bFGF, 10 ng/ml LIF, 100 U/ml penicillin, 100 μg/ml  streptomycin |  |
| ESC | HD: SI-187 (51 CAG), SIVF020 HD (48 CAG),  SIVF018 (46 CAG),  Huez2.3 (44 CAG),  VUB05 (44 CAG),  SIVF017 (40 CAG),  WT: SA-01, VUB01, H9, huES24, WT4 | Manual dissection  (every 5 – 7 days) | Cultivation  inactivated MEF layer;  medium: DMEM/F12, glutamax,  20% KO serum replacement, 1 mM NEAA, 1% penicillin/  streptomycin, 0.55 mM β-mercaptoethanol, 5 ng/ml  recombinant human FGF2 (all from Invitrogen) | [76] |
| NSC | Derived from ESC [76] | 0.05% trypsin (Invitrogen) (every 2-5 days, up to 25 passages) | Differentiation  poly-L-ornithine/laminin (Sigma);  medium: N2B27 Invitrogen) with 5 ng/ml FGF2 (Invitrogen), 300 ng/ml Noggin (Peprotech), 20 µM SB431542 (Tocris Biosciences) | [76] |
|  |  |  | Cultivation  poly-L-ornithine/laminin (Sigma);  medium: N2B27 with 10 ng/ml FGF2, 10 ng/ml EGF (R&D systems), 10 ng/ml BDNF (Peprotech) |  |
| iPSC | Isogenic lines  HD: HD-iPS4 (78 CAG),  Control line: genetically corrected  C116 iPSC (21 CAG)  C127 (20 CAG) reprogramming  OSKM (retrovirus) | 1 mg/ml collagenase (Invitrogen) | Cultivation  inactivated MEF layer;  medium: KO DMEM/F12 (Invitrogen) with 20% KO serum replacement (Invitrogen), 2.58 mM L-glutamine (Invitrogen),  1x NEAA(Invitrogen), 15.4 mM HEPES (Invitrogen), 0.5 mM β-mercaptoethanol (Invitrogen), 100 U/ml penicillin (Invitrogen), 100 μg/ml streptomycin (Cellgro), 4 ng/ml bFGF (Peprotech) | [24] |
|  |  |  | Cultivation  matrigel (BD);  medium: ES medium conditioned by MEFs |  |
| NSC | Derived from iPSC [26]  iPSC (Coriell):  HD:  #GM09197 (180 CAG),  # n/a (109 CAG),  #GM03621 (60 CAG),  WT:  #GM02183 (33 CAG),  # n/a (21 CAG), reprogramming  OSKM + Nanog, Lin28 (lentivirus) or OSKM (episomal) | chopping technique (every 7 days) | Cultivation  Polyhema;  medium: Stemline (Sigma) with 100 ng/ml bFGF (Chemicon), 100 ng/ml  EGF (Chemicon), and 5 mg/ml heparin (Sigma) | [26] |
| neurons | Derived from NSC [26] | - | Differentiation step 1  laminin or aggregation in NIM (1% N2 in DMEM:F12);  medium: 20 ng/ml BDNF (Peprotech 450-02) for 2 days, BDNF, rhShh (R&D 1845-  SH), and Dkk1 (100 ng/ml; R&D 1096-DK-010) for 21 days, 0.5 mM dbcAMP (Sigma D0260), 0.5 mM valproic acid (Sigma P4546)  . | [26] |
|  |  |  | Differentiation step 2  poly-L-ornithine/laminin,  neural differentiation medium:  70% DMEM, 30% Hams F12, 1X B27, 1% penicillin/streptomycin (Invitrogen), 200 mM ascorbic acid (Sigma), 1 mM cAMP |  |
| neurons | Derived from iPSC [33]:  HD-iPSC-A1 (43 CAG),  HD-iPSC-A7 (43 CAG),  HD-iPSC-B16 (43 CAG),  WT: CON-iPSC-1,  CON-iPSC-5  reprogramming  OSKM (Fugene 6, Roche, lentivirus/retrovirus) | - | Differentiation  Step 1: Embryoid bodies to NSC  medium (Neurobasal): DMEM/F12 with  1x N2, 1% NEAA, 1 mM sodium pyruvate,  2 mM L-glutamine and 20 ng/ml bFGF (all from Invitrogen), 10 μM SB431542, 100 nM LDN193189 | [33] |
|  |  |  | Step 2: NSC to neurons  Matrigel;  medium: for 4 weeks DMEM/F12 with  0.5X N2, 0.5X B27, 1% NEAA, 0.5 mM sodium pyruvate,  2 m L-glutamine, 10 ng/ml bFGF, for 4-6 weeks with 1X B27, 1% NEAA,  2 mM L-glutamine |  |
| neurons | Derived from iPSC [25] reprogramming  OSKM (lentivirus) | - | Differentiation  embryoid bodies to NSC and NSC to neurons  medium (K-1): DMEM/F12 (PanEco), 1x N2 (Life Technologies), 80 ng/ml Noggin (Peprotech), 4 ng/ml bFGF (Peprotech), 8 μM SB431542 (Stemgent), 2 μM Dorsomorphin (Stemgent);  medium (K-2): DMEM/F12, 1x N2, 80 ng/ml Noggin, 4 ng/ml bFGF, 0.65 μM Purmorphamine (Stemgent),  medium (K-3): DMEM/F12, 1x N2, 10 ng/ml bFGF, 0.65 μM Purmorphamine, 4 μM Forskolin (Stemgent),  medium (K-4): Neurobasal-A (Life Technologies), 1x B27 (Life Technologies), 10 ng/ml BDNF (Peprotech), 4 μM Forskolin | [25] |
| ESC | HD: GENEA046 (45 CAG),  WT: GENEA019 | N/A | N/A | [34] |
| neurons | Derived from ESC [34]  HD: GENEA020 (48 CAG),  GENEA018 (46 CAG),  GENEA046 (45 CAG),  WT: GENEA019, GENEA 23, GENEA029 | N/A | Differentiation  medium (Neurobasal)(Life Technologies): with 100 ng/mL Noggin and 100 ng/mL BDNF (R&D Systems) | [34] |
| iPSC | HD: from Coriell  GM04281 (68 CAG)  GM01187 (47 CAG)  WT: from ATCC  551-8  reprogramming OSKM (pMIG vector, lentivirus) | N/A | Cultivation  medium: 80% DMEM/F12, 20% KO Serum Replacement, 10 ng/ml bFGF,  1 mM L-glutamine, 100 μM NEAA, 100 μM 2-mercaptoethanol, 50 U/ml penicillin, and 50 mg/ml streptomycin | [47] |
| neurons | Derived from iPSC [47] | - | Differentiation Step 1: iPSC to NSC  Medium: for 8 days G-MEM with 10% KSR,  2 mM glutamine, 1 mM pyruvate, 0.1 mM NEAA, 0.1 mM 2-ME, with PA6 cells  For 6 days with N2, 100 μM tetrahydrobiopterin, 200 μM ascorbate, 2 mM glutamine, 1 mM pyruvate, 0.1 mM NEAA, 0.1 mM β-mercaptoethanol | [47] |
|  |  |  | Differentiation  Step 2: NSC to neurons  poly-L-ornithine/fibronectin;  medium: G-MEM with 10% KSR, 2 mM glutamine, 1 mM pyruvate, 0.1 mM NEAA, 0.1 mM 2-ME, 20 ng/ml BDNF (R&D Systems) |  |

* CAG repeat length – for allele with mutation, ** - information about source of reagents is provided when available in original research work.
